# *Betula mcallisteri* sp. nov. (sect. *Acuminatae*, Betulaceae), a new diploid species overlooked in the wild and in cultivation, and its relation to the widespread *B. luminifera*

**DOI:** 10.3389/fpls.2023.1113274

**Published:** 2023-05-23

**Authors:** Huayu Zhang, Junyi Ding, Norbert Holstein, Nian Wang

**Affiliations:** ^1^State Forestry and Grassland Administration Key Laboratory of Silviculture in Downstream Areas of The Yellow River, College of Forestry, Shandong Agricultural University, Tai’an, Shandong, China; ^2^Mountain Tai Forest Ecosystem Research Station of State Forestry and Grassland Administration, College of Forestry, Shandong Agricultural University, Tai’an, Shandong, China; ^3^Department of Life Sciences, The Natural History Museum, London, United Kingdom

**Keywords:** *Betula*, botanic garden, introgression, RAD-seq, polyploid

## Abstract

Taxa are traditionally identified using morphological proxies for groups of evolutionarily isolated populations. These proxies are common characters deemed by taxonomists as significant. However, there is no general rule on which character or sets of characters are appropriate to circumscribe taxa, leading to discussions and uncertainty. Birch species are notoriously hard to identify due to strong morphological variability and factors such as hybridization and the existence of several ploidy levels. Here, we present evidence for an evolutionarily isolated line of birches from China that are not distinguishable by traditionally assumed taxon recognition proxies, such as fruit or leaf characters. We have discovered that some wild material in China and some cultivated in the Royal Botanic Gardens Edinburgh, formerly recognized as *Betula luminifera*, differ from other individuals by having a peeling bark and a lack of cambial fragrance. We use restriction site-associated DNA sequencing and flow cytometry to study the evolutionary status of the unidentified *Betula* samples to assess the extent of hybridization between the unidentified *Betula* samples and typical *B. luminifera* in natural populations. Molecular analyses show the unidentified *Betula* samples as a distinct lineage and reveal very little genetic admixture between the unidentified samples and *B. luminifera*. This may also be facilitated by the finding that *B. luminifera* is tetraploid, while the unidentified samples turned out to be diploid. We therefore conclude that the samples represent a yet unrecognized species, which is here described as *Betula mcallisteri*.

## Introduction

Recognizing and naming entities is a fundamental step to manage and use biodiversity. The central unit to handle biodiversity is the species. These units are used to make generalized statements and predictions about its individual members, e.g., about behaviors, reactions to environment, edibility, dangers, or requirements for cultivation.

There are long-standing theoretical debates about what a species is, leading to multiple species concepts (Mayden, 1997; de Queiroz, 1998). Most basically, species are considered as a group of populations with active or at least potential ongoing free gene flow along generations—coined the “biological species concept” (Mayr, 1942). Historically, however, due to the lack of other methods, species have been defined as groups of morphologically similar individuals with significant differences to other groups. Which characters are used to distinguish these different morphological species, or morphospecies, is up to each evaluating researcher (Holstein and Luebert, 2017). However, there is no generalized rule as to which (set of) characters and what degree of similarity fulfill the requirements of a coherent and predictable unit, or “species.” Once one or more characters deemed to distinguish a group from others are found, a representative specimen—the type—would be chosen to define the name of this group in addition to a description or diagnosis (Turland et al., 2018). This methodology, called taxonomy, would serve to create names for these groups.

Many names that represent species were based on the observation of only few individuals or even fragments of individuals. The observation of more individuals often reveals the full morphological scope and may blur and put initial species circumscriptions and the application of names into question. However, since different names have been created using different proxies, species concepts may be incompatible with each other, causing discussions about which (set of) characters might serve best to circumscribe a species. Many species were even described based on morphological characters that turned out to be less reliable, as they vary considerably across environments (Wang et al., 2014a; Beatty et al., 2016). Occasional or localized interspecific gene flow may give rise to hybrids or, further along through introgression, blur morphological species boundaries, which further complicates species delimitation (Tovar-Sánchez and Oyama, 2004; Bardy et al., 2011; Castillo-Mendoza et al., 2019; Beeler et al., 2020; Wehenkel et al., 2020). Some hybrids possess a combination of parental morphological characters and sometimes were regarded as species (Barnes and Dancik, 1985), while hybridization can also lead to such true species by establishing reproductive barriers, e.g., though polyploidization (Ferguson and Sang, 2001; Rieseberg, 2001). In plants, diploid and tetraploid close relatives usually have a strong reproductive isolation as triploids are mostly sterile, impeding interspecific gene flow (Levin, 1975; Fowler and Levin, 1984; Rieseberg, 2001; Husband and Sabara, 2004; Roccaforte et al., 2015). However, polyploids exhibit a mosaic of parental characters or even new ones (Rieseberg et al., 2003; Ahmed et al., 2019). In consequence, the detection of “true species” and analysis of their evolution can be challenging in groups in which the reproductive barriers of once isolated genetic lines are low, e.g., in *Salix* (Marchenko and Kuzovkina, 2022).

To delineate species more reliably, an integrated approach has been increasingly adopted, combining morphological, molecular, and (or) cytogenetic data (Fujita et al., 2012; Alors et al., 2016; Newton et al., 2020; Giacò et al., 2022; Wang et al., 2022). The advantage of using an integrated approach for species delimitation is that it overcomes the subjective species concepts (Hey, 2006; Hausdorf, 2011; Carstens et al., 2013) . Phylogenomic approaches generate a multitude of characters that are usable for analyzing complex questions. The large number of single-nucleotide polymorphisms (SNPs) obtained from next-generation genotyping allows for robustly inferring the phylogenetic position and ploidy level of a diploid or tetraploid species (Cariou et al., 2013; Zohren et al., 2016; Grewe et al., 2017).

*Betula* (birch) includes approximately 65 species and subspecies, which are broadly distributed across the Northern Hemisphere (Ashburner and McAllister, 2013). Birches are well known for their frequent hybridization (Anamthawat-Jónsson and Tómasson, 1999; Thórsson et al., 2010; Wang et al., 2014b; Tsuda et al., 2017; Bona et al., 2018; Ding et al., 2021), due to wind pollination, self-incompatibility, and lack of complete reproductive barriers (Ashburner and McAllister, 2013). *Betula* species exhibit substantial morphological variation. For example, bark color varies considerably among *B. pendula* and *B. pubescens* populations (Tarieiev et al., 2019). In addition, *Betula* species have a series of ploidies, ranging from the diploid to dodecaploid (Ashburner and McAllister, 2013; Wang et al., 2016). Consequently, *Betula* has a very tough taxonomy, and species misidentification often occurs (Wang et al., 2016).

We came across some individuals during fieldwork in the Qinling-Daba Mountains in central China, which have similar fruits and leaves and an overlapping phenology with the widespread species *Betula luminifera* H.J.P.Winkl. (Winkler, 1904). However, these individuals have a peeling bark, whereas *B. luminifera* has a smooth bark according to the Flora of China (Li, 1979; Li and Skvortsov, 1999). We also noticed that these samples lacked the typical wintergreen fragrance when the bark was damaged, a character commonly found in specimens identified as *B. luminifera*. We termed these individuals as “unidentified samples” hereafter. We also looked at cultivated material of “*B. luminifera*” in botanical gardens and found an accession (#19933472) at the Royal Botanic Gardens Edinburgh, which shares the characters of the unidentified samples. We integrated multiple lines of evidence to resolve the taxonomic status of the “unidentified sample” and then investigated the amount of gene flow with *B. luminifera*.

The aims of our study were (1) to resolve the taxonomic status of the “unidentified sample”, (2) to explore if *B. luminifera* #19933472 and the “unidentified sample” are conspecific, and (3) to investigate the extent of gene flow between the “unidentified sample” and *B. luminifera*. To this end, we collected 38 individuals of the “unidentified sample” and 48 individuals of the *B. luminifera* samples from 4 and 13 natural populations, respectively.

## Materials and methods

### Species identification and sampling

**Figure 1:**
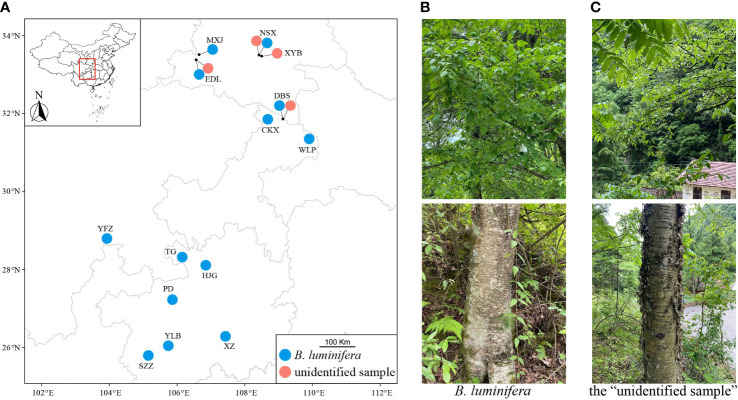
Sampling localities **(A)**, female catkins and barks of *B luminifera* **(B)**, and the “unidentified sample” **(C)**.

We collected *B. luminifera* and the “unidentified sample” between May and September of 2019, 2020, and 2021 from 13 and 4 populations, respectively (**Figure 1A**). Adjacent samples were separated by ~20 m. *Betula luminifera* was identified based on a morphological description according to Flora of China and the monograph of the *Betula* species (Li and Skvortsov, 1999; Ashburner and McAllister, 2013). Key features to recognize *B. luminifera* include a single pendulous female catkin in raceme, a smooth bark, a fruiting period between April and June, and a strong fragrance from fresh cambial tissues (**Figure 1B**). To further accurately identify *B. luminifera*, we included samples from Guizhou province where *B. luminifera* commonly occurs. We grouped individuals as the “unidentified sample”, based on the following characteristics: a single pendulous female catkin in raceme, a peeled bark, a fruiting period between April and June, and no obvious fragrance from fresh cambial tissues (**Figure 1C**). A GPS system (UniStrong) was used to record the coordinate points of each population. Detailed sampling information is provided in **Table S1**.

We measured a subset of the material that we collected (deposited in SDAU) and herbarium specimens from BM and K [abbreviations according to Thiers updated continuously (Thiers, 2023)] with bark samples or sufficient annotations of the bark morphology.

### DNA extraction, sequencing, and read filtering

We extracted high-quality DNA from cambial tissues following a modified 2× CTAB (cetyltrimethylammonium bromide) protocol (Wang et al., 2013). Extracted DNA was assessed with 1.0% agarose gels.

### ITS sequencing

We amplified nuclear ribosomal (nr)ITS for 25 individuals of the “unidentified sample” using primers ITS4 (White et al., 1990) and ITSLeu (Baum et al., 1998). Reactions were performed following Hu et al. (2019). PCR products were sequenced at Tsingke Company (Qingdao, China). ITS sequences were deposited at NCBI with GenBank accession numbers OP263695-OP263719.

### RADseq and read filtering

A total of 86 DNA samples were selected for RADseq using an Illumina HiSeq 2500 and 150-bp pair-end sequencing with the restriction enzyme *Pst*I (Personalbio company, Shanghai, China). RADseq data of four samples of closely related *Betula* species in Wang et al. (2021) were included for population genomic analyses, representing one each of *B. alnoides*, *B. cylindrostachya*, *B. hainanensis*, and *B. luminifera* #19933472. Raw data were trimmed using Trimmomatic (Bolger et al., 2014) in paired-end mode. Reads with a quality of below 20 within the sliding window of 5 bp and unpaired reads were discarded. Then, a SLIDINGWINDOW step was performed to discard reads shorter than 40 bp.

### Read mapping and SNP calling

Filtered reads of each sample were mapped to the whole genome sequence of *B. pendula* (Salojärvi et al., 2017) using the BWA-MEM v.0.7.17-r1188 algorithm in BWA with default parameters (Li and Durbin, 2009). Alignments were converted to sorted and indexed bam files using SAMtools v1.8 (Li et al., 2009). The MarkDuplicates tool and HaplotypeCaller from GATK v 4.1.4 were used to mark duplicates and call genotypes for each sample, respectively (Mckenna et al., 2010; Depristo et al., 2011). The GenomicsDBImport was used to merge the gVCF files into a combined VCF file, which was used for joint genotyping using the GenotypeGVCFs tool. The SNPs were filtered using a mapping quality (MQ) threshold of 40, a variant confidence (QUAL) of 30, a normalized QUAL score (QD) of 2.0, a maximum symmetric odds ratio (SOR) of 3.0, a minimum depth (DP) of 5 and a maximum depth of 200, a maximum probability of strand bias (FS) of 60.0, an excess heterozygosity (ExcessHet) of 54.69, a minimum *Z*-score of read mapping qualities (MQRankSum) of −12.5, and a position bias (ReadPosRankSum) of −8.0. The SelectVariants filtering tool was applied to select SNPs present in at least 90 of the samples. BCFtools v1.8 was used to remove SNPs within a 50-kb window with *r*^2^ > 0.5 to reduce linkage disequilibrium and then was used to remove SNPs with a minimum allele frequency (MAF) ≤ 0.02 (Li, 2011). All sequences were deposited in the NCBI-Sequence Read Archive (SRA) repository under the BioProjectID PRJNA871086.

### Population genomic analyses

A principal component analysis (PCA) was performed on the SNPs of *B. luminifera*, the “unidentified sample”, and the four samples of closely related *Betula* species using the “adegenet” R package 2.1.1 (Jombart, 2008). The SNPs were also analyzed in ADMIXTURE v1.3.0 (Alexander and Lange, 2011), setting K from 1 to 10 with 20 replicates for each K value. Cross-validation error estimation was performed in order to assess the most suitable value of K (Alexander and Lange, 2011). Replicate runs were aligned and visualized in pong v1.4.9 with the greedy algorithm (Behr et al., 2016).

### Inference of ploidy level based on flow cytometry and SNPs

We performed flow cytometry following Wang et al. (2022) for three accessions of the “unidentified sample” collected from the populations NSX and XYB. Briefly, we co-chopped fresh cambial tissues with internal standards *Solanum lycopersicum* in 0.5 ml of Extraction Buffer (Cystain PI absolute P, Partec GmbH, Germany) and then filtered them into a tube containing 1 ml of Staining Solution (Cystain PI absolute P, Partec GmbH). We added 50 µl of diluted propidium iodide (PI) to the tube and incubated samples at room temperature in the dark for ~5 min. We analyzed one replicate per sample with >5,000 nuclei measured using flow cytometry (Beckman Coulter CytoFLEX). We also inferred the ploidy of the 86 samples sequenced in this study using the method described in Zohren et al. (2016). Briefly, we plotted the distribution of the allele ratios from read counts at heterozygous sites, and we expected a peak of approximately 0.50 for a diploid, peaks of approximately 0.33 and 0.67 for a triploid, and peaks close to 0.25, 0.50, and 0.75 for a tetraploid.

### Phylogenetic analyses based on ITS, ITS secondary structure, and SNPs

Seventy additional ITS sequences from Betulaceae (Wang et al., 2016; Wang et al., 2022) were included to infer the phylogenetic position of the “unidentified sample”. A total of 95 ITS sequences and their secondary structure (ITS1 and ITS2), as previously used by Tarieiev et al. (2021), were aligned separately using BioEdit v.7.0.9.0 (Hall, 1999) with default parameters.

We included the RADseq data of 23 *Betula* taxa from a previous study (Wang et al., 2021) and of 45 samples generated in the present study for phylogenomic analysis. *Alnus inokumae* was selected as the outgroup. The SNPs were concatenated into a supermatrix with missing data below 50%. This resulted in 2,309,898 SNPs.

We analyzed the ITS alignment, the ITS1 alignment, the ITS2 alignment, and the supermatrix of SNPs separately, with a rapid bootstrap analysis under a GTR + GAMMA nucleotide substitution model in RAxML v.8.1.16 (Stamatakis, 2006). A total of 100 bootstraps and 10 searches using the ML were performed.

## Results

### Read mapping and variant calling

The individual read mappings of *B. luminifera* and the “unidentified sample” from the present study resulted in 8.8% to 95.1% of mapped reads per individual. After filtering, 340,979 variants were present in at least one individual (**Table S1**).

### Population structure

**Figure 2:**
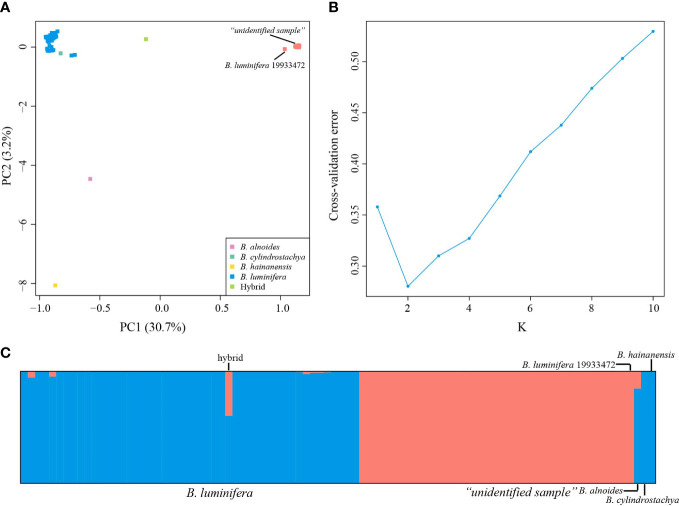
Population structure analyses based on RADseq data. **(A)** Principal component analysis (PCA) of *Betula* species of section *Acuminate* at 40,209 SNPs. **(B)** Cross-validation error calculated for Ks from 1 to 10. **(C)** Estimated genetic admixture of 90 *Betula* samples at 40,209 SNPs. Each individual is represented by a vertical line. Blue and orange represent *B luminifera* and the “unidentified sample”, respectively. Previously sequenced samples (*B. luminifera* #19933472, *B alnoides*, *B cylindrostachya*, and *B hainanensis*) are placed on the right side.

PC1 and PC2 explained 30.7% and 3.2% of the total variation, respectively (**Figure 2A**). The PCA (**Figure 2A**) results based on the 40,209 biallelic SNPs revealed a clear separation between *B. luminifera* and the “unidentified sample” on PC1. *Betula luminifera* #19933472 formed a cluster with the “unidentified sample”. A putative hybrid between *B. luminifera* and the “unidentified sample” was positioned in between. A specimen identified as *Betula cylindrostachya* formed a cluster with *B. luminifera*. *Betula alnoides* and *B. hainanensis* were separated from *B. luminifera* on PC2.

Consistent with the PCA results, admixture analyses showed the optimal K value of 2 (**Figure 2B**), corresponding to *B. luminifera* and the “unidentified sample”/*B. luminifera* #19933472. The “unidentified sample”/*B. luminifera* #19933472 formed a distinct lineage even when the K value was increased to 6 (**Figure S1**). Very little admixture was detected in the “unidentified sample” into *B. luminifera*, with the highest admixture value of 5.31% (excluding the putative hybrid). The sample EDL20-022 showed a genetic admixture of 39.76% and 60.24% of the “unidentified sample” and *B. luminifera*, respectively (**Figure 2C**).

A high level of genetic differentiation was observed between *B. luminifera* and the “unidentified sample”, with F_ST_ values among populations with at least four individuals ranging from 0.30 to 0.43. Within species, F_ST_ values ranged from 0.01 to 0.04 for *B. luminifera*. The F_ST_ value between the two populations of the “unidentified sample” was 0.02.

### Ploidy-level analyses

**Figure 3:**
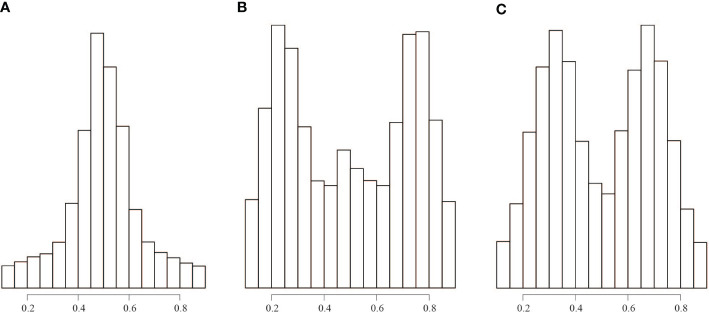
Distribution of read count ratios for heterozygous sites covered by at least 30 reads. **(A)** All the “unidentified sample” individuals. **(B)** All *B luminifera* individuals. **(C)** Sample number EDL20-022, which is a hybrid between *B luminifera* and the “unidentified sample”.

Flow cytometry analyses showed that the genome size of the three “unidentified sample” ranged from 388 to 445 M, indicating that it is diploid (**Figure S2**). The plot of the allele ratios at heterozygous sites showed that the “unidentified sample” had a peak of approximately 0.50, *B. luminifera* had peaks near 0.25, 0.50, and 0.75, and the putative hybrid between the “unidentified sample” and *B. luminifera* had peaks close to 0.33 and 0.67 (**Figures 3**, **S3**, **S4**). One *B. luminifera* individual (YLB001) had a ploidy level that remains unclear due to the low number of reads (**Figure S3**).

### Phylogenetic position of the “unidentified sample”

**Figure 4:**
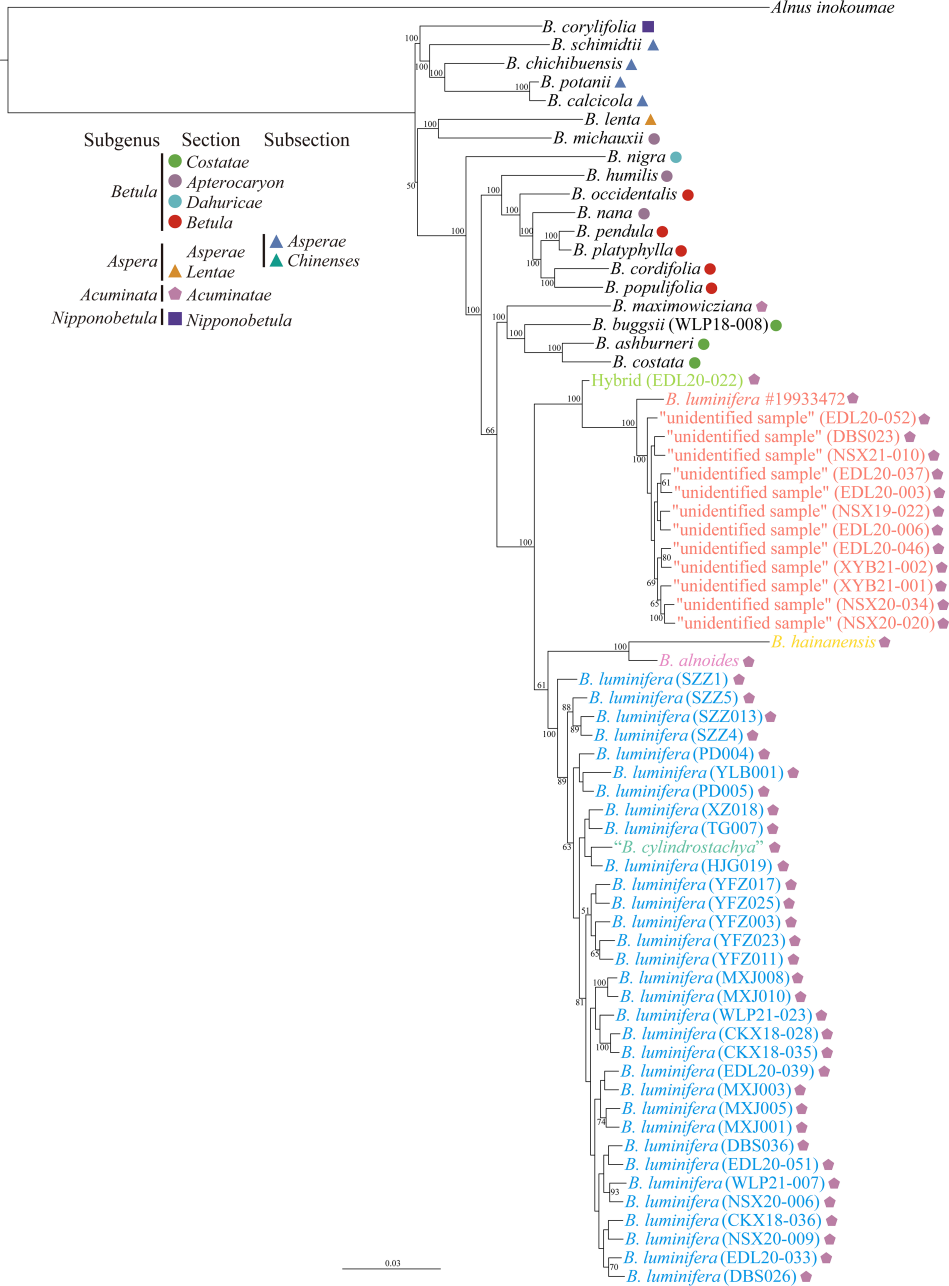
Phylogenetic tree from the maximum likelihood analysis of the “unidentified sample” based on a supermatrix of 2,309,898 SNPs. The scale bar below indicates the mean number of nucleotide substitutions per site. Species were classified according to Ashburner and McAllister (2013). Values close to branches are bootstrap percentages of >50%.

The phylogenetic tree based on ITS1 failed to resolve the phylogenetic position of the “unidentified sample”; however, based on ITS or ITS2, it showed that the “unidentified sample” tended to form a cluster, except for sample EDL20-022, which we interpret as an F_1_ hybrid between the “unidentified sample” and *B. luminifera* (**Figures S5–S7**). The phylogenetic tree based on a supermatrix of 2,309,898 SNPs revealed that the *B. luminifera* #19933472 and the “unidentified sample” formed a well-supported monophyletic clade, which is a sister to a clade of *B. luminifera*, *B. cylindrostachya*, *B. alnoides*, and *B. hainanensis* (**Figure 4**). *B. cylindrostachya* was nested into a well-supported clade of *B. luminifera* samples (**Figure 4**).

### Features of the *B. luminifera* #19933472, the “unidentified sample”, and *B. luminifera*

Features of *B. luminifera* #19933472 and the “unidentified sample” include a peeling bark (**Figure S8**), no fragrance from fresh cambial tissues, and diploidy, which are distinct from that of *B. luminifera.* We measured 16 samples of “*B. luminifera*” with a peeling bark and 6 of the “unidentified samples” and did not find any leaf, flower, or infructescence characters that could be used to distinguish peeling and non-peeling individuals (**Table 1**). Non-peeling individuals seem to have had a higher number of side veins and larger male flower scales, but the sizes were overlapping.

**Table 1:** Features of the *B. luminifera* #19933472, the “unidentified sample”, and *B. luminifera*.

	*B. luminifera*^1^ #19933472	The “unidentified sample”	*B. luminifera*
Ploidy level	Diploid	Diploid	Tetraploid
Level of fragrance	Low	Low	High
Patterns of bark peeling	Peeled along lenticels	Peeled along lenticels	Smooth
Bark color	Red–purple	From red–purple to light brown	From red–purple to gray
Female catkins	A single pendulous	A single pendulous	A single pendulous
Petiole_length [mm]	na	9–16	7–19
Leaf length × width [mm]	na	52–108 × 25–60	35–120 × 19–72
Leaf no. of side veins	na	12–18	7–14
Pedunculus of fem ale catkin [mm]	na	9–19	4–20
Mature female catkin_length × width [mm]	na	27–66 × 3–6	40–105 × 4–9
Scale_total size_length × width [mm]	na	3.2–3.5 × 1.2–1.6	2.6–5.0 × 0.7–2.0
Scale_central lobe_length × width [mm]	na	1.4–2.0 × 0.7–1.2	1.5–2.9 × 0.5–1.3
Scale_lateral lobe_length × width [mm]	na	0.1–0.4 × 0.2–0.5	0.1–0.8 × 0.1–0.6
Scale_surface	na	At base and margin with 0.3-mm-long trichomes	At base and margin with up to 0.3-mm-long trichomes
Seed size_length × width [mm]	na	1.6–2.2 × 0.8–1.1	1.0–2.0 × 0.8–1.3
Seed wing-width [mm]	na	1.1–1.7	1.1–2.1
Seed surface	na	Apically with trichomes	Apically with trichomes
Style_length [mm]	na	0.6–0.8	0.4–1.7
Anther length [mm]	na	0.7–1.1	0.8–1.4

^1^na represents unmeasured morphological traits.

## Discussion

### A new diploid species in section *Acuminatae*

Our results show that *B. luminifera* #19933472 and the “unidentified samples” with a peeling bark form a distinct genetic cluster based on admixture results at the value of K = 2 and onwards (**Figure S1**). Phylogenomic analyses strongly support them as a monophyletic clade, which is separated from smooth-barked *B. luminifera* and other species of section *Acuminatae* (**Figure 4**).

The type material of *B. luminifera* was collected from Chengkou County in the north of the municipality of Chongqing (Winkler, 1904), very much in the vicinity of our “unidentified samples” (northeastern part of Chongqing and just north of Chongqing in the southern part of Shaanxi province). Although we do not know the exact locality where the type specimen of *B. luminifera* was collected, nor is there any description of the bark in the protologue of *B. luminifera*, the current understanding of *B. luminifera* is that it has a smooth bark (Li, 1979; We et al., 1987; Li and Skvortsov, 1999; Fu et al., 2004; Ashburner and McAllister, 2013). Smooth-barked *B. luminifera* is widespread in the subtropical monsoon climate areas of China (Li and Skvortsov, 1999). While the four wild populations of the “unidentified sample” were from the Qinling-Daba Mountains (Shaanxi, NE Chongqing), *B. luminifera* #19933472 was collected from Yunnan province in southwest China. This indicates that this group may have a wider distribution, and our sampling shows that it grows even sympatrically with the smooth-barked *B. luminifera*.

All the samples with a peeling bark are found to be diploid, while the smooth-barked *B. luminifera* material from our study is tetraploid. *Betula luminifera* has been reported and considered as diploid in previous studies (Ashburner and McAllister, 2013; Wang et al., 2016; Wang et al., 2021), but the material used for that analysis by Wang et al. (2016) and Wang et al. (2021) is in fact “*B. luminifera*” #19933472. All the samples from Chengkou County in this study are found to have a smooth bark and to be tetraploid. Hence, the description of *B. luminifera* as diploid (Ashburner and McAllister, 2013) is likely incorrect. *Betula luminifera* may also have different cytotypes, like other *Betula* species, such as *B. chinensis* (Ashburner and McAllister, 2013). However, we do not find any contradiction from the correlation of ploidy, bark morphology, and clustering in the phylogeny to support different ploidy levels in smooth-barked *B. luminifera*.

Although we did not conduct pollination experiments, a difference in ploidy indirectly indicates reproductive barriers, especially between the diploid and tetraploid (Borges et al., 2012). We expect that the specimens with a peeling bark also fit the biological species concept that focuses on reproductive isolation (Mayr, 1942). We therefore propose that they are accepted as a new species, which here we name *Betula mcallisteri*. Aside from our case, a difference in ploidy has also been used to separate other closely related *Betula* species. For example, *B. pendula* and *B. pubescens* were once treated as a single species, *B. alba* (Linnaeus, 1753), and were later acknowledged to be two species, as *B. pendula* is a diploid and *B. pubescens* is a tetraploid (Tuley, 1973; Atkinson, 1992).

### Hybridization between *B. mcallisteri* and *B. luminifera*

*Betula mcallisteri* and *B. luminifera* are closely related. Given the difference in ploidy level, it can be assumed that an ancestor of the diploid *B. mcallisteri* may have been involved in the evolution of the tetraploid *B. luminifera*.

Allele sharing among closely related species is usually ascribed to incomplete lineage sorting and/or introgressive hybridization (Twyford and Ennos, 2012). Incomplete lineage sorting is an unlikely explanation for the pattern of allele sharing observed between *B. mcallisteri* and *B. luminifera*. In most allopatric populations of *B. luminifera*, no allele transfer from *B. mcallisteri* to *B. luminifera* was detected, whereas in sympatric populations, either hybrid or a low level of genetic admixture was detected (**Figure 2C**). Such a geographic signal would not be expected from an incomplete lineage sorting (Barton, 2001).

The sample EDL20-022 showed a high level of genetic admixture, which stood out from other samples. We interpret this result as a putative F_1_ hybrid. The presence of a hybrid in the population EDL where both species occur sympatrically indicates that *B. mcallisteri* hybridized with *B. luminifera*, meeting our expectation that interploidy hybridization could occur among *Betula* species (Zohren et al., 2016; Tsuda et al., 2017; Hu et al., 2019). However, introgression between *B. mcallisteri* and *B. luminifera* was not detected in this population. In contrast, in the sympatric population NSX, hybrids between *B. mcallisteri* and *B. luminifera* were not detected, but signals can be interpreted as traces of introgression (between 0.3% and 2%) from *B. mcallisteri* to *B. luminifera* (**Figure 3C**).

Asymmetric introgression from the diploid to tetraploid was also observed in other studies (Wang et al., 2014b; Clark et al., 2015; Eidesen et al., 2015; Zohren et al., 2016; Tsuda et al., 2017). However, there is very little introgression from *B. mcallisteri* to *B. luminifera*, with the highest admixture value of 5.3%, much lower than the level of introgression from *B. nana* to *B. pubescens*, which shows the highest admixture value of 16.9% (Zohren et al., 2016). One possible explanation is that triploids between *B. mcallisteri* and *B. luminifera* may produce less viable gametes than triploids between *B. nana* and *B. pubescens*, which were reported to produce a small proportion of viable gametes (Anamthawat-Jónsson et al., 2021). Alternatively, we may have failed to sample the introgressed individuals. The fact that we did not detect hybrid swarms indicates a strong reproductive barrier between *B. mcallisteri* and *B. luminifera*, likely due to a difference in ploidy.

### Taxonomic issues in section *Acuminatae*

Multiple lines of evidence support that the “unidentified samples” and the cultivated *B. luminifera* #19933472 represent a yet undescribed species, here described as *B. mcallisteri*. *Betula mcallisteri* belongs to *Betula* sect. *Acuminatae* (Regel, 1865), with its long pendulous female catkins and widely winged seeds with wings wider than the nutlet (Skvortsov, 1997; Ashburner and McAllister, 2013).

*Betula mcallisteri* has a single pendulous female catkin, like *B. luminifera*, and both can be distinguished from most of the other species of section *Acuminatae*, which have three to five pendulous female catkins in a raceme, such as *B. alnoides*, *B. fujianensis*, and *B. hainanensis* (Zeng et al., 2008; Zeng et al., 2014). However, single female catkins also occur in *B. cylindrostachya* (Ashburner and McAllister, 2013), including the type material (Wallich, 1830; Kumar et al., 2022), although two or three catkins are more common.

The number of female catkins being one or two may therefore not be a reliable character for species delimitation in members of section *Acuminatae*. For example, Hugh McAllister identified a sample with two female catkins as *B. cylindrostachya*, and it was nested in a clade of *B. luminifera* (**Figure 4**). This is consistent with Skvortsov’s view that *B. cylindrostachya* is most closely related to *B. luminifera* (Skvortsov, 1997). Indeed, in some populations, *B. luminifera* sporadically has two female catkins in a raceme [e.g., *S. Chen 481* (K), *S.J. Zhang 4493* (NAS00284530), and *S.K. Lai 2577* (KUN0529329, LBG00108762); personal communication with Jie Zeng], and *B. cylindrostachya* occasionally has a single female catkin (Ashburner and McAllister, 2013). However, *B. cylindrostachya* is considered to be tetraploid and to have a peeling and fragrant bark (Ashburner and McAllister, 2013; Kumar et al., 2022), which is in contrast to Skvortsov’s hypothesis.

*Betula mcallisteri* is diploid, and several other samples identified as *B. alnoides*, *B. cylindrostachya*, and *B. luminifera* are tetraploid, based on genome size estimation or inference from reads covering heterozygous SNPs (**Figures S2–S4**). *Betula alnoides* was once considered as diploid (Mehra and Sareen, 1973), but it was found to be tetraploid based on the chromosome counting of samples from several populations (personal communication with Hugh McAllister). We did not see the voucher of the material that was used by Mehra and Sareen. Their assumption that the material must have been *B. alnoides* because *B. cylindrostachya* (misspelled as “*cylindrostachys*”) would be only occurring in the Eastern Himalayas is puzzling, because *B. cylindrostachya* was actually described from the Kumaon district (Wallich, 1830; Kumar et al., 2022), the very same area where their voucher is from. It could mean that (1) there are diploid members of section *Acuminatae* in India (assuming that the authors studied local material), and (2) the name *B. cylindrostachya* may have been misapplied currently. However, only *B. alnoides* and *B. cylindrostachya* from section *Acuminatae* are currently accepted. Both species are easily confused and rather weakly differentiated (Ashburner and McAllister, 2013). How the tetraploid species in section *Acuminatae* evolved, speciation after polyploidization or independent polyploidizations, and how they are morphologically differentiated require further investigation.

We could not find any traditional characters in flower, fruit, or leaf morphology to distinguish *B. mcallisteri* from *B. luminifera*. Only bark smooth vs. peeling (**Figures 1B, C**) and cambial fragrance turned out to be suitable macromorphological and field characters. The number of leaf side veins and male flower scale sizes are different but overlapping (**Table 1**). Since most herbarium material does not contain any sample or description of the bark, our sampling is rather small. It is likely that these two characters would turn out to be even less helpful in distinguishing the two groups if more material from other regions and habitats were to be examined. This leaves natural history collections with a peculiar problem: herbarium collections commonly do not include or even mention bark characters. Even though the bark is often ornamental in *Betula* (Ashburner and McAllister, 2013), it is often ignored or imprecisely annotated when collecting birches. This renders the majority of “*B. luminifera*” collections as indeterminable. Our results exemplify why it is important to document taxa more extensively, including bark samples, or at least to add information using photography. The call to collect more extensive information for a better understanding of the organism is not new (Webster, 2017), especially since new questions about interactions of plants with their environment are given more research focus (Schindel and Cook, 2018). In birches, however, it may make the difference between determinable and indeterminable natural history collections.

Our discovery of *B. mcallisteri* being in cultivation at RBGE for 30 years is another nice example that botanic gardens may grow undiscovered species. Such cases may exist for other plant species with poorly understood taxonomy. A broad knowledge of morphological variation from wild populations is important in examining species delimitation. Botanic gardens play an important role in cultivating and conserving plant species (O'donnell and Sharrock, 2017) and provide a venue for conducting various research activities (Chen and Sun, 2018). While getting suspected new species into cultivation is not unusual, only few examples of material being cultivated for a long time and turning out to be new to science are known, e.g., *Oncidium herrenhusanum* (Schlumpberger and Königer, 2011). Also, the recently discovered *Victoria boliviana* was initially suspected to be *Victoria amazonica*, and living material helped to resolve its species status (Smith et al., 2022). Living collections in botanic gardens can supplement valuable information for taxonomy and species discovery. However, our finding that the RBGE accession #19933472 was used to determine the ploidy level of *B. luminifera* (Wang et al., 2016; Wang et al., 2021), despite the morphological difference of the bark from what is commonly understood as *B. luminifera*, may serve as a reminder that *pars pro toto* statements for species could lead to incorrect assumptions. It also stresses the importance of the voucher material and detailed descriptions.

### Taxonomic treatment

**Figure 5:**
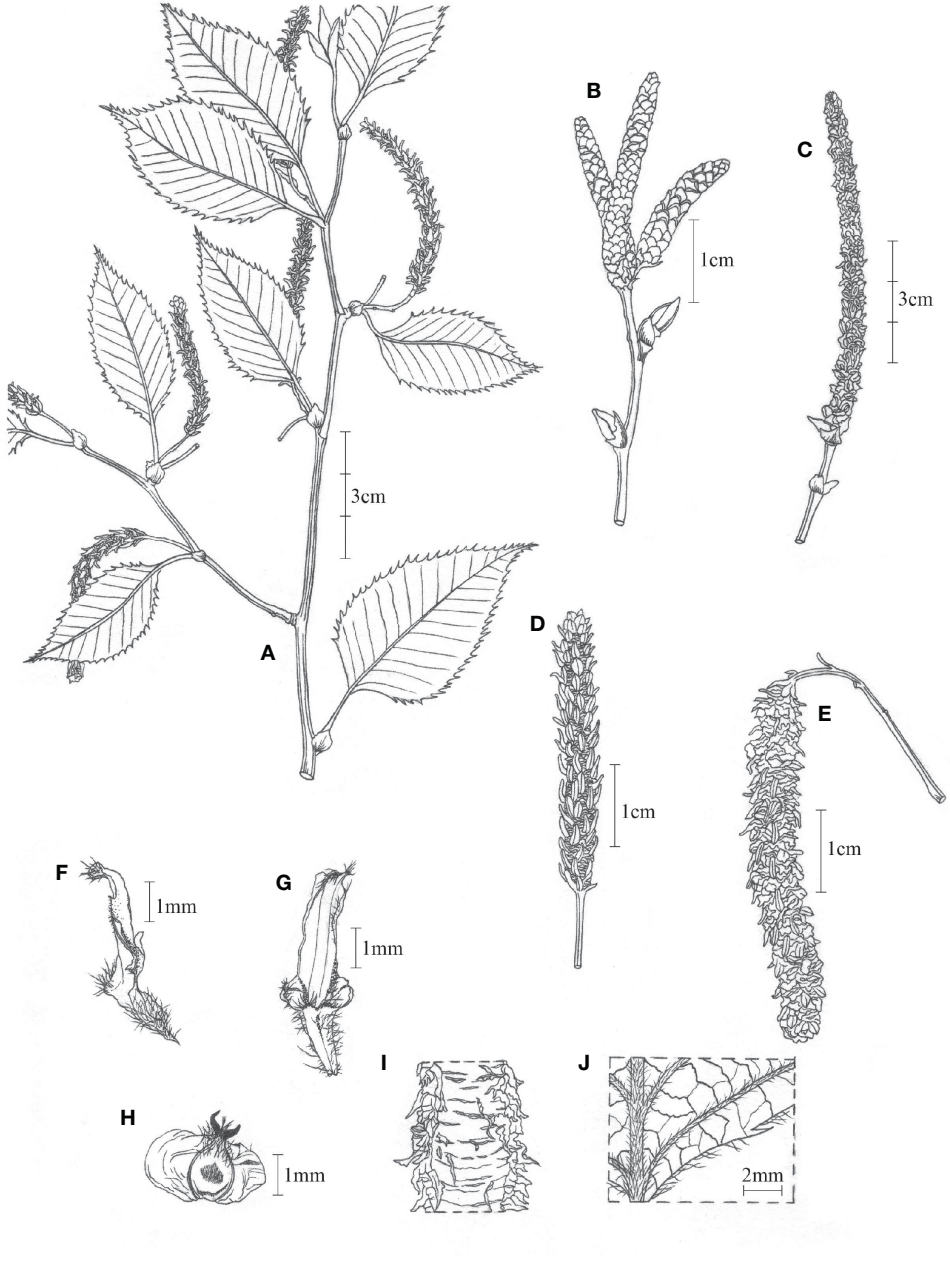
*Betula mcallisteri*. **(A)** Specimen with female catkins; **(B)** male catkins, immature; **(C)** male catkins, mature; **(D)** female catkin, immature; **(E)** female catkin, mature; **(F)** side view of fruiting catkin scale; **(G)** fruiting catkin scale, outer face; **(H)** mature seed; **(I)** bark; **(J)** adaxial surface of leaf blade.

*Betula mcallisteri*, Nian Wang & Holstein, sp. nov (**Figure 5**).

#### Diagnosis

*Betula mcallisteri* is a tree species with single pendular female catkins, just like *B. luminifera* (rarely also 2 catkins), but in contrast to other species in section *Acuminatae* (two or more female catkins, single ones in *B. cylindrostachya* only exceptionally). It differs from *B. luminifera* by having a bark that peels off in horizontal strips instead of a smooth, non-peeling bark. Also, its damaged bark does not have a noticeable fragrance of oil of wintergreen (methyl salicylate), while *B. luminifera* is noticeably fragrant when the bark is damaged.

Type: China. Shaanxi: Mian County, elev. ca. 1,600–2,000 m, 33.4° N, 106.6° E, 6 May 2021, *Nian Wang* DEDL001 (**Figure S9**) (holotype IBSC0895006; isotypes HIB0216644, IBSC0895007, HITBC0083551, KUN1571214, K, and PE).

#### Description

Single trunked tree to 30 m high. Trunk up to 60 cm in diameter; fresh bark red–brown, peeling along transverse lenticels into persistent and tattered sheets. Leaves one to three per node, accessory leaves often smaller. Petiole (3–)7–16 mm, densely beset with short trichomes but glabrescent, sometimes with reddish or whitish glands. Leaves ovate, 52–108 × 25–60 mm, 12–18 side veins, all running into a tooth on the margin. Leaf tip short apiculate, base round to widely cuneate. Leaf margin double-serrate. Upper lamina darker than below, with silky trichomes on lamina between the side veins, glabrescent. Lower lamina with red glands, silky trichomes along the veins, glabrescent. Male catkins in clusters of three to four, pendent when matured, ~10 × 0.3 cm, length/breadth ratio ~30:1. Male flower bracts scale-like, 1.5–1.8 × 1.2–1.5 mm, margin ciliate. Anthers 0.7–1.1 mm long. Female catkins with a 9- to 16-mm-long peduncle. Immature female catkins green, born singly, pendent, up to 5 cm long, and 6.5 mm broad including the scales. Fruiting catkins, borne singly, long cylindrical, up to ~100 × 6–8 mm at maturity; bracts scale-like, 3.2–3.5 × 1.2–1.6 mm, trilobate with central lobe dominating; adaxially at base with a tuft of up to 0.5 mm long trichomes, margin sometimes ciliate, surfaces more or less glabrous; central lobe narrowly oblong or rhombic, 1.4–2.0 × 0.7–1.2 mm; lateral lobes 0.1–0.4 × 0.2–0.5 mm short rounded to triangulate. Seed 1.6–2.2 × 0.8–1.1 mm, wing 1.1–1.7 mm wide on each side, translucent, styles 0.6–0.8 mm; seed surface hairy on apical half.

#### Distribution and habitat

*Betula mcallisteri* occurs in the Qinling-Daba Mountains in central China, according to our field survey, and in Yunnan province, SW China, based on the collection information from *B. luminifera* #19933472. *Betula mcallisteri* grows sympatrically with *B. luminifera* at an altitude between 1,400 and 2,100 m. *Betula mcallisteri* and *B. luminifera* have no altitudinal separation. We found abundant *B. mcallisteri* individuals in EDL and NSX populations, with 16 and 30 samples confirmed as *B. mcallisteri*, respectively.

#### Conservation status

Habitat destruction and logging are common issues for the survival of woody species. The area where population NSX is located once underwent severe tree logging before China introduced the Natural Forest Protection Program and the Slope Land Conversion Program in 1998 (Delang and Wang, 2013). Since then, the area has been under strict protection, and logging is totally prohibited. Different from the NSX population, the area where the EDL population was located is near a village and was cut through by a road allowing for car traveling. The finding of a cultivated specimen originating from Yunnan suggests that the species is more widely distributed but overlooked. We therefore do not see any indication to assume that *B. mcallisteri* requires a conservation status and propose to categorize it as least concern (LC).

#### Etymology

*Betula mcallisteri* is named after Hugh A. McAllister, a botanist from the Institute of Integrative Biology, University of Liverpool, for his devotion to research on the genus *Betula*. The Chinese name of *B. mcallisteri* is “陕南桦” (shǎn nán huà; literally: Shannan birch).

## Data availability statement

The datasets presented in this study can be found in online repositories. The names of the repository/repositories and accession number(s) can be found in the article/**Supplementary Material**.

## Author contributions

NW conceived the project; NW and JD collected the samples; HZ and NH performed the experiments; HZ, JD and NH analyzed the data; HZ, NH and NW wrote the manuscript. All authors contributed to the article and approved the submitted version.
