# Supplementary material for: Betula mcallisteri sp. nov. (sect. Acuminatae, Betulaceae), a new diploid species overlooked in the wild and in cultivation, and its relation to the widespread B. luminifera
Source: Front Plant Sci. 2023 May 23;14:1113274. doi: 10.3389/fpls.2023.1113274 (PMC10268003; doi:10.3389/fpls.2023.1113274)
Supplement: Supplementary file 1 [file DataSheet_1.pdf]

Supplementary Material

Figure S1 Admixture results at K values from 2 to 6 based on 40,209 SNPs.
Figure S2 Genome size estimation of the three individuals of the "unidentified sample". Red and blue peaks represent the "unidentified sample" and the internal standard (tomato), respectively.
Figure S3 Plots of read count ratios for heterozygous sites covered by at least 30 reads for *B. luminifera*.
Figure S4 Plots of read count ratios for heterozygous sites covered by at least 30 reads for the "unidentified sample".
Figure S5 Phylogenetic tree from the maximum-likelihood analysis of the "unidentified sample" using ITS sequences.
Figure S6 Phylogenetic tree from the maximum-likelihood analysis of the "unidentified sample" using ITS1 sequences.
Figure S7 Phylogenetic tree from the maximum-likelihood analysis of the "unidentified sample" using ITS2 sequences.
Figure S8 Pictures of *Betula luminifera* #19933472 from the Royal Botanic Garden in Edinburgh. (A) fruit; (B) male catkins; (C) and (D) bark.
Figure S9 A picture of an isotype specimen of *B. mcallisteri* (originally labelled as DEDL001).
Table S1 Detailed information on samples used in the present study.

Figure S1 Admixture results at K values from 2 to 6 based on 40,209 SNPs.

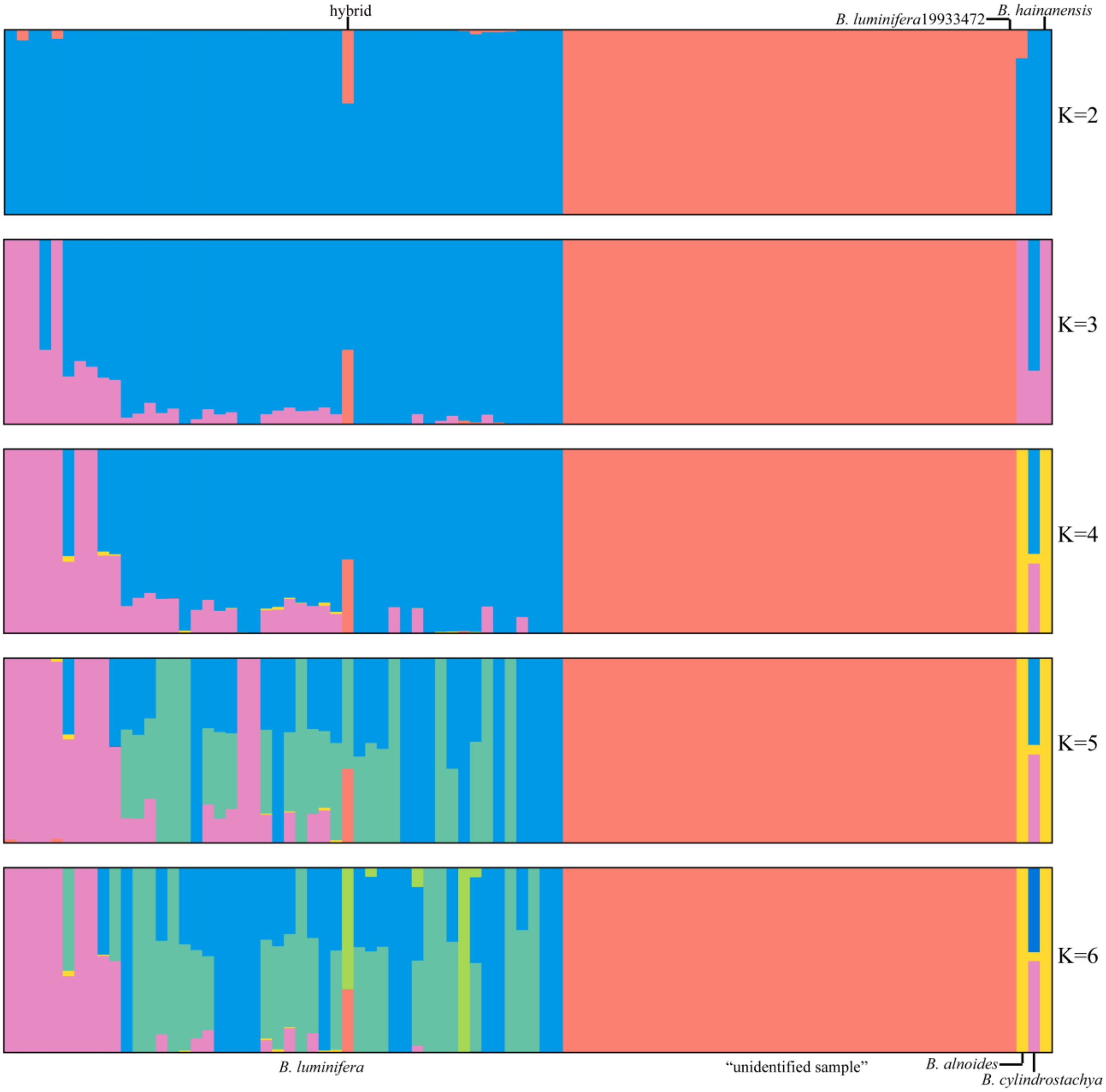

Figure S2 Genome size estimation of three individuals of the “unidentified sample”. Red and blue peaks represent the “unidentified sample” and the internal standard (tomato), respectively.

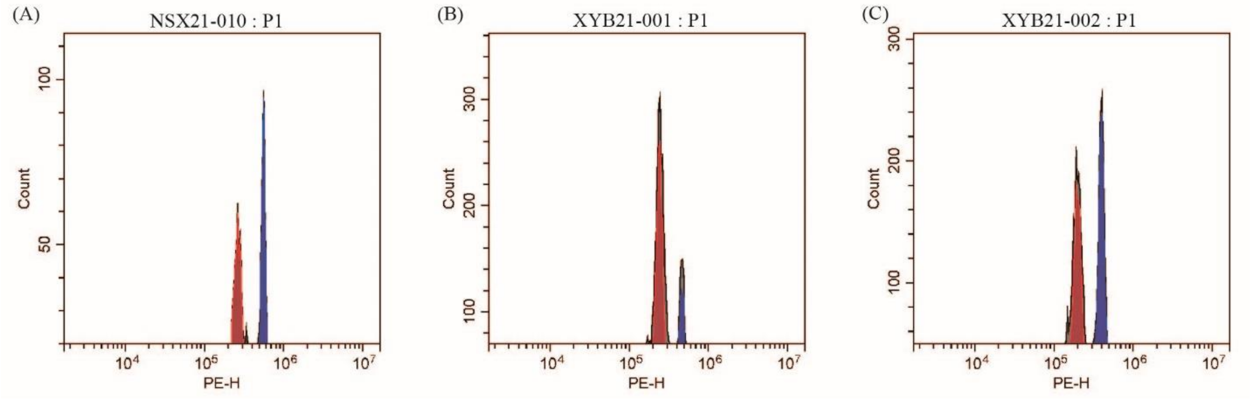

Figure S3 Plots of read count ratios for heterozygous sites covered by at least 30 reads for *B. luminifera*.

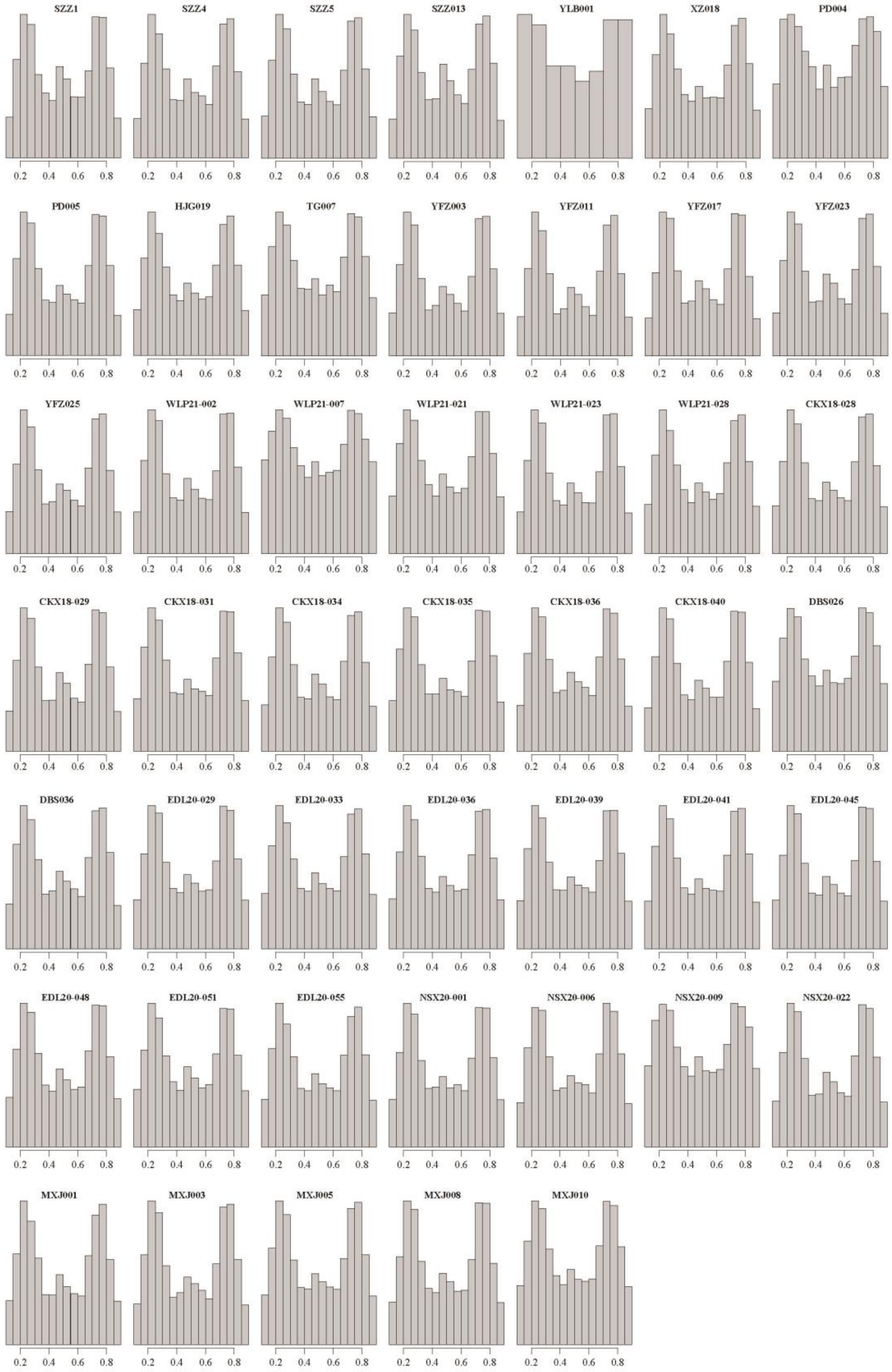

Figure S4 Plots of read count ratios for heterozygous sites covered by at least 30 reads for the "unidentified sample".

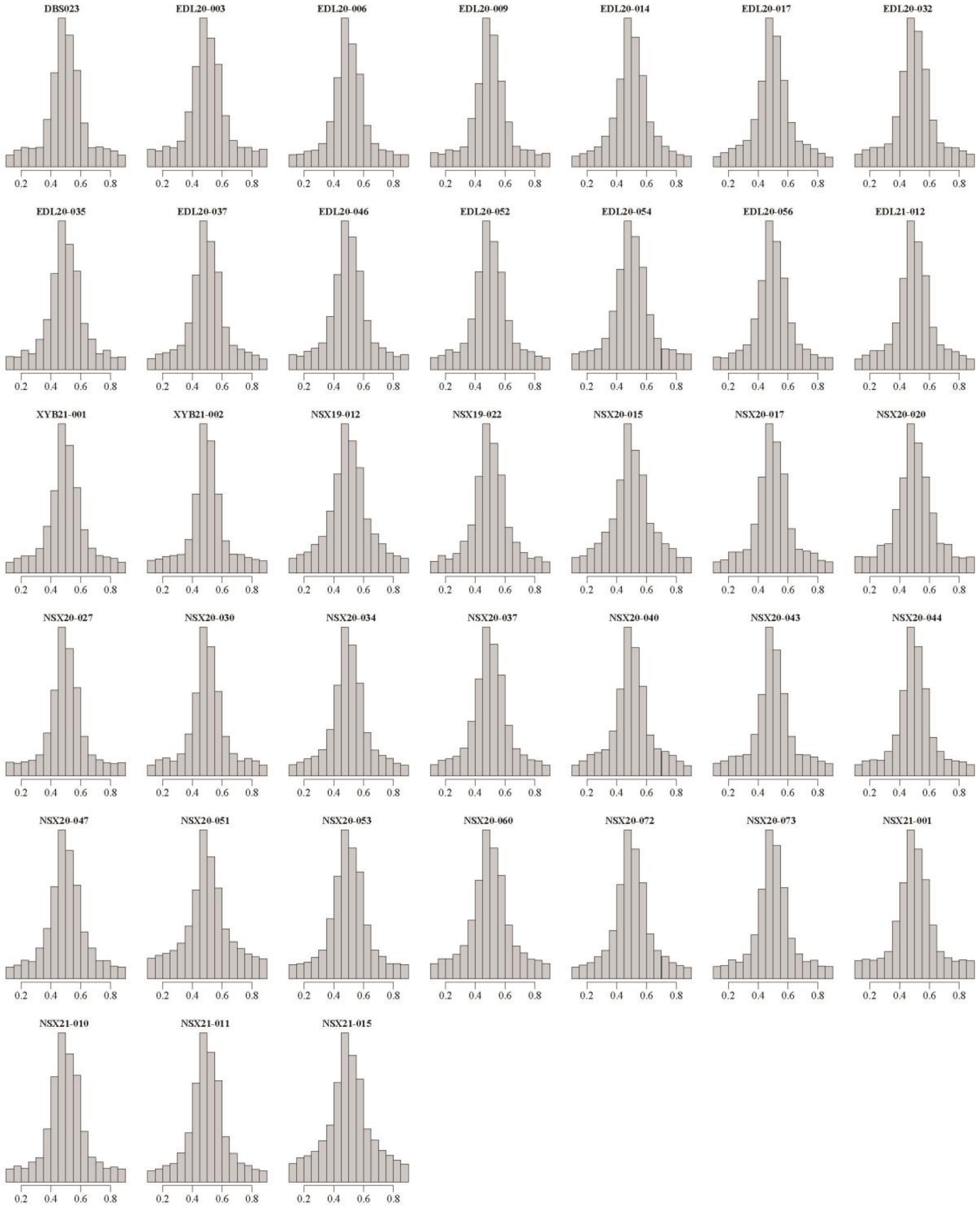

Figure S5 Phylogenetic tree from the maximum-likelihood analysis of the “unidentified sample” using ITS sequences.

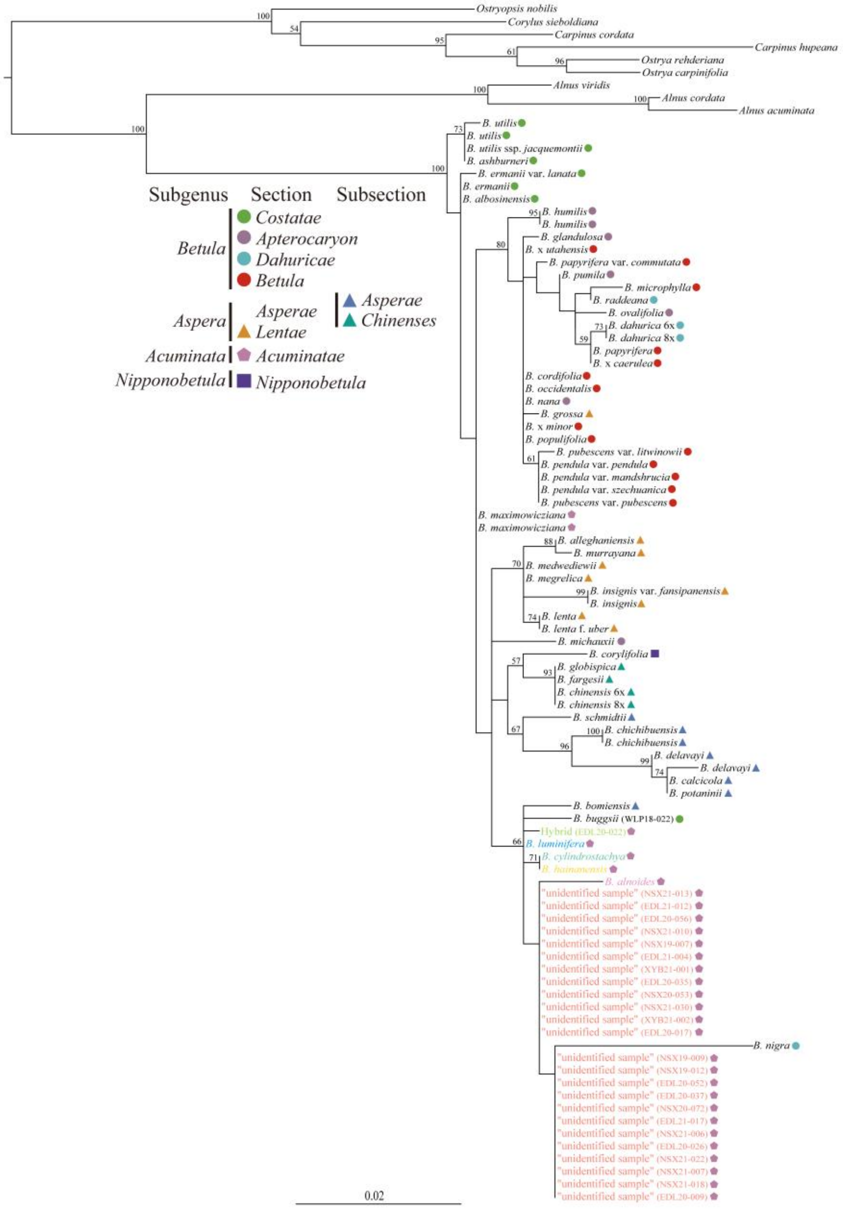

Figure S6 Phylogenetic tree from the maximum-likelihood analysis of the “unidentified sample” using ITS1 sequences.

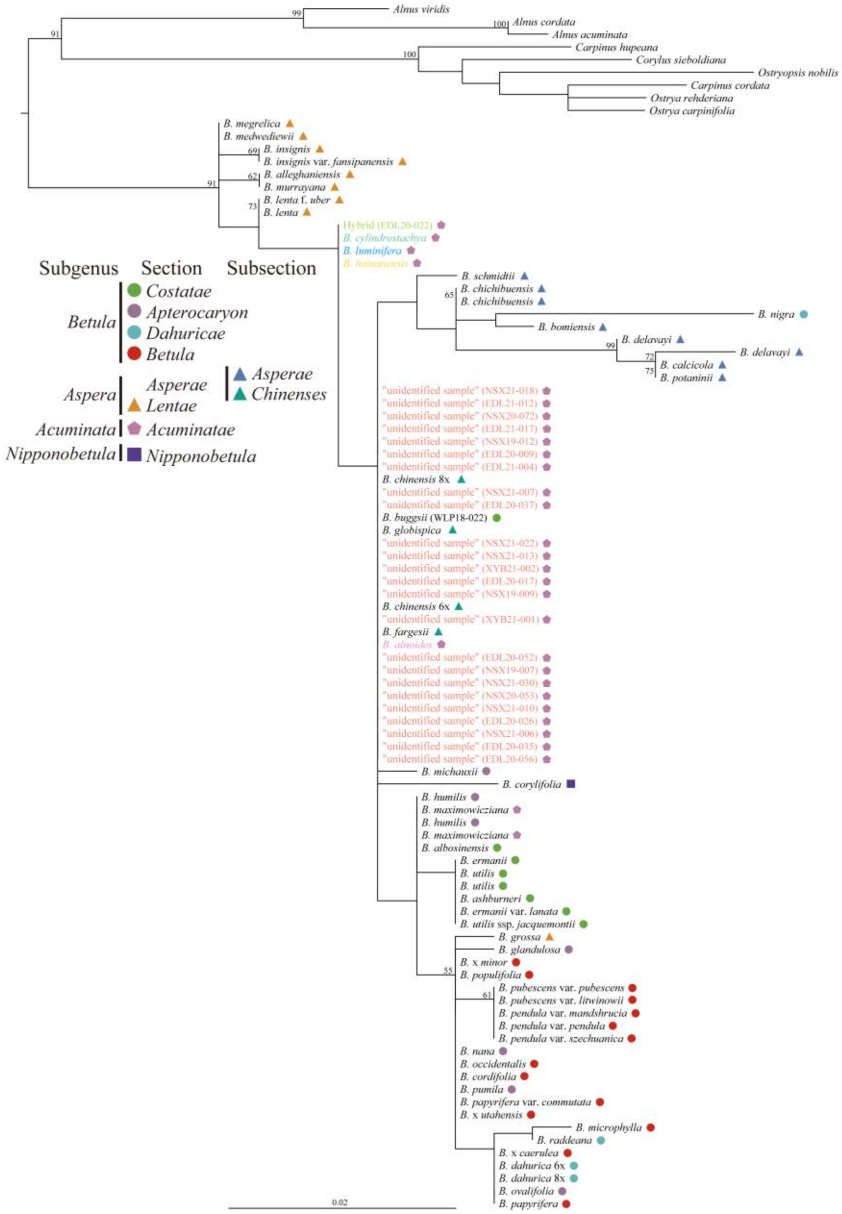

Figure S7 Phylogenetic tree from the maximum-likelihood analysis of the “unidentified sample” using ITS2 sequences.

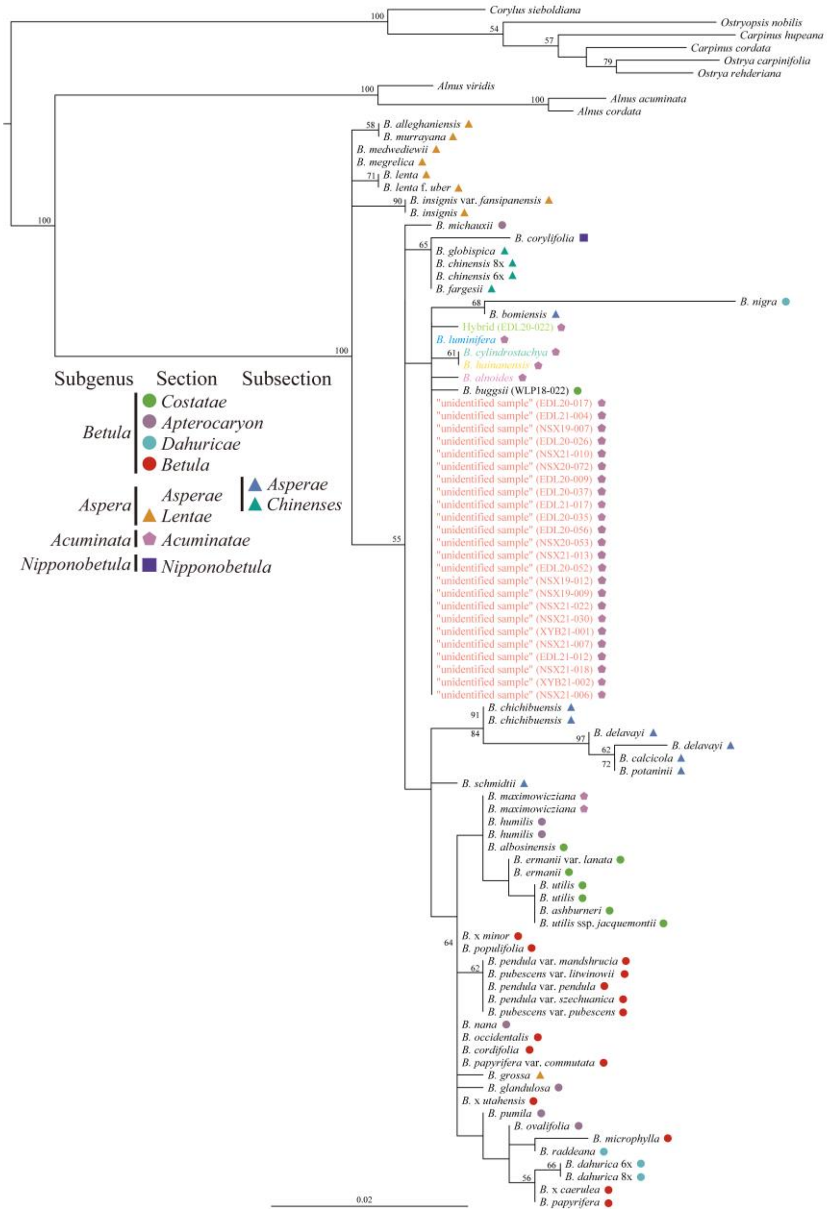

Figure S8 Pictures of *Betula luminifera* #19933472 from the Royal Botanic Garden in Edinburgh. (A) fruit; (B) male catkins; (C) and (D) bark.

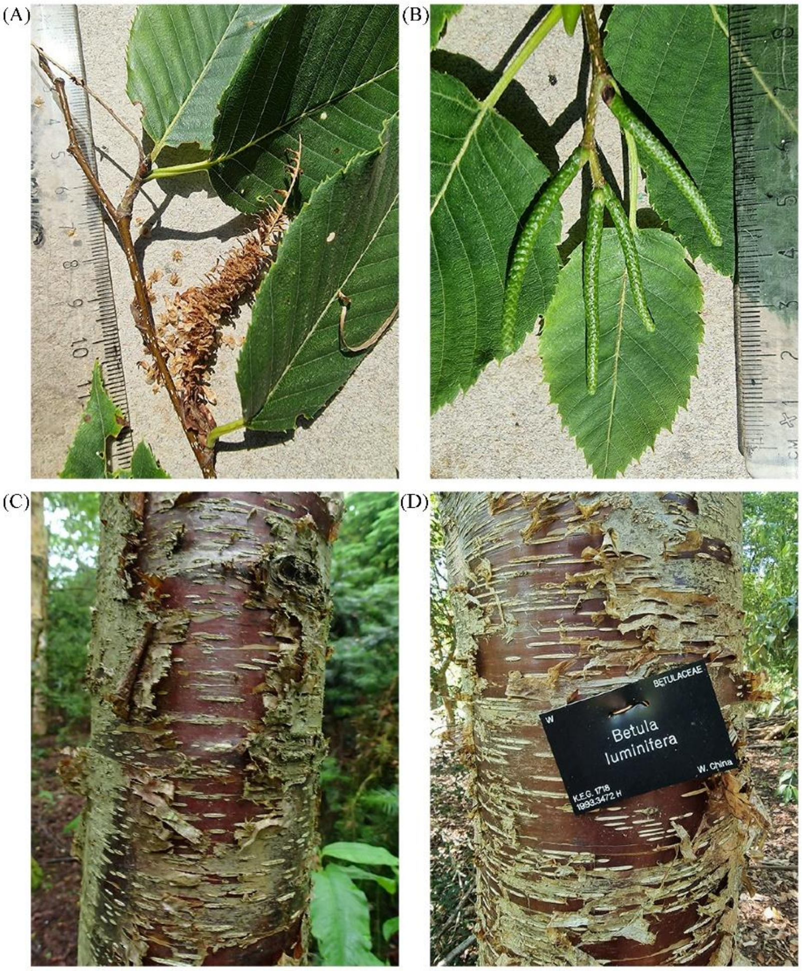

Figure S9 A picture of an isotype specimen of *B. mcallisteri* (originally labelled as DEDL001).

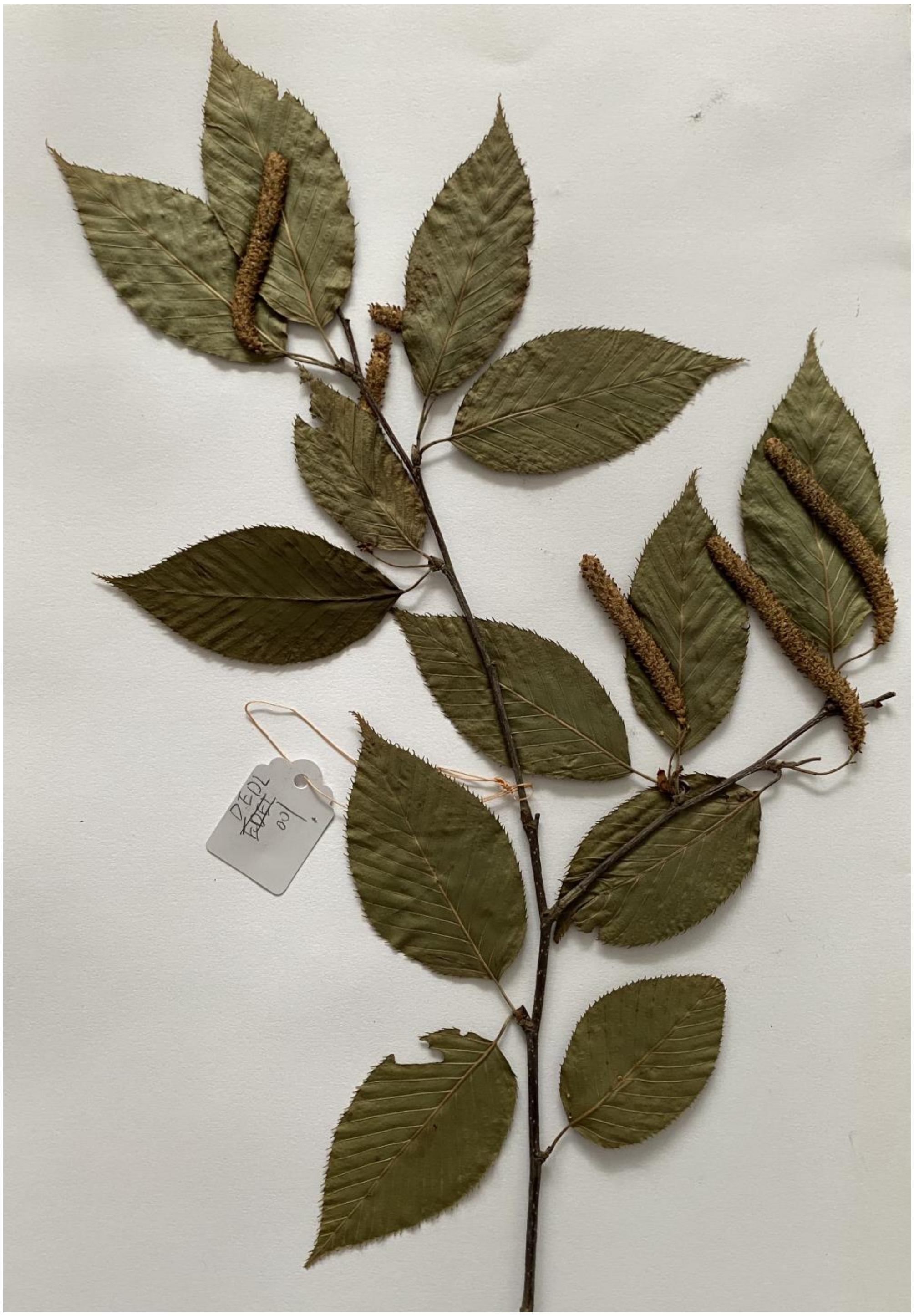

Table S1 Detailed information on samples used in the present study.

Individuals	Species[1]	Latitude (°N)	Longitude (°E)	Clean reads	Mapped reads	Mapped rate (%)	Location	Note[2]
SZZ013	*B. luminifera*	25.79	105.15	15,408,170	14,251,142	92.49	Qinglong County, Guizhou Province	SZZ013
SZZ1	*B. luminifera*	25.79	105.15	19,326,092	17,331,575	89.68	Qinglong County, Guizhou Province	SZZ1
SZZ4	*B. luminifera*	25.79	105.15	14,925,864	12,969,418	86.89	Qinglong County, Guizhou Province	SZZ4
SZZ5	*B. luminifera*	25.79	105.15	16,725,832	14,405,742	86.13	Qinglong County, Guizhou Province	SZZ5
YLB001	*B. luminifera*	26.04	105.74	31,662,030	2,789,213	8.81	Zhenning County, Guizhou Province	YLB001
XZ018	*B. luminifera*	26.28	107.43	21,255,620	9,323,543	43.86	Qiannan, Guizhou Province	XZ018
PD004	*B. luminifera*	27.23	105.86	18,219,898	6,122,000	33.60	Pudi, Guizhou Province	PD004
PD005	*B. luminifera*	27.23	105.86	15,754,046	13,448,460	85.37	Pudi, Guizhou Province	PD005
HJG019	*B. luminifera*	28.11	106.85	13,780,112	11,023,752	80.00	Tongzi County, Guizhou Province	HJG019
TG007	*B. luminifera*	28.32	106.15	23,345,108	9,050,503	38.77	Xishui County, Guizhou Province	TG007
YFZ003	*B. luminifera*	28.79	103.93	22,307,656	21,010,707	94.19	Muchuan County, Sichuan Province	YFZ003
YFZ011	*B. luminifera*	28.79	103.93	19,629,442	18,612,178	94.82	Muchuan County, Sichuan Province	YFZ011
YFZ017	*B. luminifera*	28.79	103.93	24,627,126	23,127,137	93.91	Muchuan County, Sichuan Province	YFZ017
YFZ023	*B. luminifera*	28.79	103.93	19,984,834	17,540,593	87.77	Muchuan County, Sichuan Province	YFZ023
YFZ025	*B. luminifera*	28.79	103.93	20,559,120	18,391,947	89.46	Muchuan County, Sichuan Province	YFZ025
WLP21-002	*B. luminifera*	31.35	109.90	16,577,240	13,139,252	79.26	Wushan County, Chongqing City	GYZ002
WLP21-007	*B. luminifera*	31.35	109.90	13,470,328	12,471,919	92.59	Wushan County, Chongqing City	GYZ007
WLP21-021	*B. luminifera*	31.35	109.90	22,883,706	15,261,146	66.69	Wushan County, Chongqing City	YJC012_2
WLP21-023	*B. luminifera*	31.35	109.90	24,504,488	19,754,450	80.62	Wushan County, Chongqing City	YJC014
WLP21-028	*B. luminifera*	31.35	109.90	19,522,140	14,535,074	74.45	Wushan County, Chongqing City	YJC019
CKX18-028	*B. luminifera*	31.85	108.68	15,039,260	14,199,252	94.41	Chengkou County, Chongqing City	QYS028
CKX18-029	*B. luminifera*	31.85	108.68	18,695,080	16,346,646	87.44	Chengkou County, Chongqing City	QYS29
CKX18-031	*B. luminifera*	31.85	108.68	13,295,132	11,292,091	84.93	Chengkou County, Chongqing City	QYS031
CKX18-034	*B. luminifera*	31.85	108.68	14,437,658	13,441,155	93.10	Chengkou County, Chongqing City	QYS034
CKX18-035	*B. luminifera*	31.85	108.68	14,052,940	12,833,204	91.32	Chengkou County, Chongqing City	QYS35
CKX18-036	*B. luminifera*	31.85	108.68	17,231,644	15,771,033	91.52	Chengkou County, Chongqing City	QYS36
CKX18-040	*B. luminifera*	31.85	108.68	13,581,650	12,293,962	90.52	Chengkou County, Chongqing City	QYS040

DBS026	*B. luminifera*	31.86	109.13	16,758,432	15,290,270	91.24	Chengkou County, Chongqing City	DBS026
DBS036	*B. luminifera*	31.86	109.13	14,316,708	13,047,364	91.13	Chengkou County, Chongqing City	DBS036
EDL20-029	*B. luminifera*	33.38	106.56	14,737,340	13,188,110	89.49	Mian County, Shaanxi Province	EDL029
EDL20-033	*B. luminifera*	33.38	106.56	14,037,378	12,722,634	90.63	Mian County, Shaanxi Province	EDL033
EDL20-036	*B. luminifera*	33.38	106.56	16,739,198	15,565,052	92.99	Mian County, Shaanxi Province	SX002
EDL20-039	*B. luminifera*	33.38	106.56	14,756,148	13,829,648	93.72	Mian County, Shaanxi Province	SX005
EDL20-041	*B. luminifera*	33.38	106.56	19,233,980	12,910,878	67.13	Mian County, Shaanxi Province	SX007
EDL20-045	*B. luminifera*	33.38	106.56	15,986,882	14,909,076	93.26	Mian County, Shaanxi Province	SX011
EDL20-048	*B. luminifera*	33.38	106.56	14,081,002	12,597,227	89.46	Mian County, Shaanxi Province	SX014
EDL20-051	*B. luminifera*	33.38	106.56	13,027,046	12,039,137	92.42	Mian County, Shaanxi Province	SX017
EDL20-055	*B. luminifera*	33.38	106.56	13,108,438	12,202,004	93.09	Mian County, Shaanxi Province	SX021
NSX20-001	*B. luminifera*	33.50	108.42	12,456,436	11,440,999	91.85	Ningshan County, Shaanxi Province	PHL001
NSX20-006	*B. luminifera*	33.50	108.42	13,833,224	12,792,080	92.47	Ningshan County, Shaanxi Province	PHL006
NSX20-009	*B. luminifera*	33.50	108.42	22,466,280	20,851,292	92.81	Ningshan County, Shaanxi Province	PHL009
NSX20-022	*B. luminifera*	33.50	108.42	17,597,920	16,388,975	93.13	Ningshan County, Shaanxi Province	PHL022
MXJ001	*B. luminifera*	33.51	106.65	16,521,680	15,459,277	93.57	Ningshan County, Shaanxi Province	MXJ001
MXJ003	*B. luminifera*	33.51	106.65	18,007,306	16,852,362	93.59	Ningshan County, Shaanxi Province	MXJ003
MXJ005	*B. luminifera*	33.51	106.65	20,351,920	15,079,954	74.10	Ningshan County, Shaanxi Province	MXJ005
MXJ008	*B. luminifera*	33.51	106.65	19,891,046	18,513,609	93.08	Ningshan County, Shaanxi Province	MXJ008
MXJ010	*B. luminifera*	33.51	106.65	16,295,246	14,959,510	91.80	Ningshan County, Shaanxi Province	MXJ010
DBS023	Unidentified	31.86	109.13	18,470,430	17,308,390	93.71	Chengkou County, Chongqing City	DBS023
EDL20-003	Unidentified	33.38	106.56	16,566,072	15,489,626	93.50	Mian County, Shaanxi Province	EDL003
EDL20-006	Unidentified	33.38	106.56	16,633,850	15,786,411	94.91	Mian County, Shaanxi Province	EDL006
EDL20-009	Unidentified	33.38	106.56	18,657,068	17,702,009	94.88	Mian County, Shaanxi Province	EDL009
EDL20-014	Unidentified	33.38	106.56	17,040,046	15,192,931	89.16	Mian County, Shaanxi Province	EDL014
EDL20-017	Unidentified	33.38	106.56	14,098,230	13,308,248	94.40	Mian County, Shaanxi Province	EDL017
EDL20-022	Unidentified	33.38	106.56	15,843,810	14,731,193	92.98	Mian County, Shaanxi Province	EDL022
EDL20-026	Unidentified	33.38	106.56				Mian County, Shaanxi Province	EDL026
EDL20-032	Unidentified	33.38	106.56	15,435,784	13,926,691	90.22	Mian County, Shaanxi Province	EDL032
EDL20-035	Unidentified	33.38	106.56	13,612,162	12,820,783	94.19	Mian County, Shaanxi Province	DEDL002 (SX001)

EDL20-037	Unidentified	33.38	106.56	15,210,572	14,136,421	92.94	Mian County, Shaanxi Province	DEDL001 (SX003)
EDL20-046	Unidentified	33.38	106.56	14,843,744	14,043,880	94.61	Mian County, Shaanxi Province	SX012
EDL20-052	Unidentified	33.38	106.56	13,566,732	12,789,635	94.27	Mian County, Shaanxi Province	SX018
EDL20-054	Unidentified	33.38	106.56	12,956,516	12,258,724	94.61	Mian County, Shaanxi Province	SX020
EDL20-056	Unidentified	33.38	106.56	15,331,162	14,510,529	94.65	Mian County, Shaanxi Province	SX022
EDL21-004	Unidentified	33.38	106.56				Mian County, Shaanxi Province	DEDL004
EDL21-012	Unidentified	33.38	106.56	26,951,064	13,781,877	51.14	Mian County, Shaanxi Province	DEDL012
EDL21-017	Unidentified	33.38	106.56				Mian County, Shaanxi Province	DEDL017
XYB21-001	Unidentified	33.47	108.50	29,327,080	27,650,130	94.28	Ningshan County, Shaanxi Province	DXYB001 (XYC004)
XYB21-002	Unidentified	33.47	108.50	31,562,674	29,246,050	92.66	Ningshan County, Shaanxi Province	DXYB002 (XYC001)
NSX19-007	Unidentified	33.50	108.42				Ningshan County, Shaanxi Province	NSX007
NSX19-009	Unidentified	33.50	108.42				Ningshan County, Shaanxi Province	NSX009
NSX19-012	Unidentified	33.50	108.42	17,780,376	16,667,704	93.74	Ningshan County, Shaanxi Province	NSX012
NSX19-022	Unidentified	33.50	108.42	13,145,524	12,472,356	94.88	Ningshan County, Shaanxi Province	NSX022
NSX20-015	Unidentified	33.50	108.42	12,276,032	11,494,207	93.63	Ningshan County, Shaanxi Province	PHL015
NSX20-017	Unidentified	33.50	108.42	13,731,578	12,997,801	94.66	Ningshan County, Shaanxi Province	PHL017
NSX20-020	Unidentified	33.50	108.42	14,636,686	13,815,501	94.39	Ningshan County, Shaanxi Province	PHL020
NSX20-027	Unidentified	33.50	108.42	19,510,274	18,285,370	93.72	Ningshan County, Shaanxi Province	PHL027
NSX20-030	Unidentified	33.50	108.42	20,032,244	18,846,778	94.08	Ningshan County, Shaanxi Province	PHL030
NSX20-034	Unidentified	33.50	108.42	15,742,126	14,918,637	94.77	Ningshan County, Shaanxi Province	PHL034
NSX20-037	Unidentified	33.50	108.42	17,196,280	16,296,191	94.77	Ningshan County, Shaanxi Province	PHL037
NSX20-040	Unidentified	33.50	108.42	16,878,752	15,926,965	94.36	Ningshan County, Shaanxi Province	PHL040
NSX20-043	Unidentified	33.50	108.42	18,332,120	17,434,357	95.10	Ningshan County, Shaanxi Province	PHL043
NSX20-044	Unidentified	33.50	108.42	17,207,740	16,314,835	94.81	Ningshan County, Shaanxi Province	PHL044
NSX20-047	Unidentified	33.50	108.42	19,605,296	14,004,724	71.43	Ningshan County, Shaanxi Province	PHL047
NSX20-051	Unidentified	33.50	108.42	23,931,550	22,182,907	92.69	Ningshan County, Shaanxi Province	PHL051
NSX20-053	Unidentified	33.50	108.42	12,895,952	12,174,301	94.40	Ningshan County, Shaanxi Province	PHL053
NSX20-060	Unidentified	33.50	108.42	12,920,410	12,215,698	94.55	Ningshan County, Shaanxi Province	PHL060
NSX20-072	Unidentified	33.50	108.42	16,922,580	15,784,852	93.28	Ningshan County, Shaanxi Province	PHL072
NSX20-073	Unidentified	33.50	108.42	17,435,630	16,394,245	94.03	Ningshan County, Shaanxi Province	PHL073

NSX21-001	Unidentified	33.50	108.42	19,480,110	18,288,096	93.88	Ningshan County, Shaanxi Province	DHDT001
NSX21-006	Unidentified	33.50	108.42				Ningshan County, Shaanxi Province	DHDT006
NSX21-007	Unidentified	33.50	108.42				Ningshan County, Shaanxi Province	DHDT007
NSX21-010	Unidentified	33.50	108.42	13,393,748	12,598,298	94.06	Ningshan County, Shaanxi Province	DHDT010
NSX21-011	Unidentified	33.50	108.42	12,376,610	10,491,929	84.77	Ningshan County, Shaanxi Province	DHDT011
NSX21-013	Unidentified	33.50	108.42				Ningshan County, Shaanxi Province	DHDT013
NSX21-015	Unidentified	33.50	108.42	19,771,890	18,470,913	93.42	Ningshan County, Shaanxi Province	DHDT015
NSX21-018	Unidentified	33.50	108.42				Ningshan County, Shaanxi Province	DHDT018
NSX21-022	Unidentified	33.50	108.42				Ningshan County, Shaanxi Province	DHDT024
NSX21-030	Unidentified	33.50	108.42				Ningshan County, Shaanxi Province	DHDT031_2

[1]Unidentified represents the "unidentified sample"; [2]The labels listed here represent the original labels used during field collection. Four samples have two different labels as they were collected twice and were assigned different labels.
